# Is it necessary to use tobramycin-dexamethasone eye ointment prophylactically in eyes at the end of intraocular surgery?

**DOI:** 10.1186/s12886-020-01476-z

**Published:** 2020-05-27

**Authors:** Wei Zhang, Han Han, Kang Feng, Xiaohong Wang, Mei Du, Xiangda Meng, Yuanyuan Liu, Bo Huang, Rodrigo Brant, Hua Yan

**Affiliations:** 1 Tianjin Eye Hospital, Tianjin Key Lab of Ophthalmology and Visual Science, Tianjin Eye Institute, Clinical College of Ophthalmology Tianjin Medical University, Tianjin, China; 2 Department of Ophthalmology, Tianjin Medical University General Hospital, No. 154 Anshan Road, Tianjin, 300052 China; 3 Department of Ophthalmology, Peking University Third Hospital, Beijing, China; 4 Laboratory of Molecular Ophthalmology, Tianjin Medical University, Tianjin, China; 5 Department of Pharmacology and Tianjin Key Laboratory of Inflammation Biology, School of Basic Medical Sciences, Tianjin Medical University, Tianjin, China; 6 Department of Ophthalmology, University of Mississippi Medical Center, Jackson, MS 39216 USA; 7 Department of Ophthalmology and Visual Sciences, Federal University of São Paulo, São Paulo, 04023-062 Brazil

**Keywords:** Vitrectomy, Cataract extraction, Glaucoma, Antibiotics, prophylactic, Endophthalmitis

## Abstract

**Background:**

There are no data available regarding the complications associated with using antibiotic ointment at the end of intraocular surgery. This study aimed to explore the necessity of using ocular tobramycin-dexamethasone prophylactically at the end of intraocular surgery.

**Methods:**

This was a retrospective cohort study of patients who received intraocular surgery at Tianjin Medical University General Hospital from January 2015 to December 2017. The patients were grouped according to whether they received tobramycin-dexamethasone eye ointment or not after surgery. The Tobramycin dexamethasone eye ointment was sampled to observe bacterial contamination pathogens at 0.5, 1, 1.5, 2, 2.5, 3, 6, 8, 24, 36, 48, 72, and 168 h after being opened.

**Results:**

A total of 3811 eyes in 3811 patients (mean age of 63 ± 12 years) were included: 2397 eyes that received prophylactic tobramycin-dexamethasone eye ointment and 1414 eyes that did not. The overall rate of endophthalmitis was 0.08% (3/3811) in our study, all in the eye ointment group (0.12%, 3/2397); no patients developed endophthalmitis in the non-ointment group (0%, 0/1414)(*P* = 0.184). The anterior chamber reactions 1 day after surgery were more serious in the eye ointment group compared with the non-ointment group (all *P* < 0.05), but there were no statistically significant differences at 1 month postoperatively (all *P* > 0.05). The contamination rate was 0% at all time points over 7 days.

**Conclusion:**

We did not observe a statistically significant difference in the incidence of endophthalmitis in patients with or without prophylactic tobramycin-dexamethasone eye ointment. And tobramycin-dexamethasone eye ointment seemed to increase some side effects such as eye secretions increasing and foreign body feeling.

## Background

Intraocular surgery has become more widely performed to treat a wide variety of eye diseases due to the development of surgical technology. Postoperative endophthalmitis is a rare but disastrous complication that can greatly reduce vision and even cause blindness or enucleation [1–3]. The incidence of endophthalmitis after intraocular surgery varies from 0.02 to 0.84% [4, 5].

Risk factors associated with endophthalmitis after intraocular surgery include inadequate wound closure [6], a postoperative hypotonic state [7], vitreous incarceration at a sclerotomy site [4], aqueous intraocular tamponade [8], and additional concomitant intraocular procedures [9]. Recent prophylactic trials for endophthalmitis has included preoperative antibiotic drops, conjunctival sac flushing, and antibiotic cream at the end of intraocular surgery [10]. The most common cause of endophthalmitis is Gram-positive bacteria, mainly *Staphylococcus epidermis, Staphylococcus aureus, Clostridium, and Bacillus* [11]. A trial with over 3000 cases reported that topical povidone-iodine can significantly decrease the incidence of endophthalmitis [12]. Several studies have shown that prophylactic antibiotics are useful for preventing infection, but there are no scientific guidelines to support the use of preoperative antibiotics [13]. The use of preoperative or perioperative topical antibiotics is still controversial, and the use varies widely among countries. In Sweden, prophylactic topical antibiotics are not recommended during refractive lens exchange or in high-risk patients. In Germany, prophylactic topical antibiotics are used pre- and postoperatively in all patients undergoing intraocular surgery [14]. A previous study from the European Society of Cataract and Refractive Surgeons in 2012 showed that intracameral antibiotics were used in 74% of cases, 82% of which received cefuroxime, while the others received vancomycin, moxifloxacin, or gentamicin [15]. Tobramycin-dexamethasone has been shown to be safe and effective in controlling inflammation and infection after cataract surgery [16].

The repeated use of topical antibiotics can significantly increase the possibility of colonization of the ocular surface by resistant organisms [17]. The common use of antibiotic ointment may cause super bacteria or viruses to grow and proliferate, resulting in the exacerbation of the eye condition. There are no data available regarding the complications associated with using antibiotic ointment at the end of intraocular surgery.

To explore the necessity of using topical ocular tobramycin-dexamethasone prophylactically at the end of intraocular surgery, we conducted a retrospective cohort study investigating the rate of postoperative endophthalmitis and complaints of patients who underwent intraocular surgery.

## Methods

### Patients

This was a retrospective cohort study of patients who received intraocular surgery at Tianjin Medical University General Hospital from January 2015 to December 2017. A total of 3811 eyes in 3811 patients were devided into Eye ointment group received prophylactic tobramycin-dexamethasone eye ointment(*n* = 2397) and Non-ointment group that did not (*n* = 1414) in this study (Table 1). The study was approved by the Tianjin Medical University medical ethics committee and was done in accordance with the Declaration of Helsinki. The need for individual consent was waived by the committee.

**Table 1:** Characteristics of the patients

Variable	Eye ointment group (n = 2397)	Non-ointment group (*n* = 1414)	P
Male, n (%)	997 (41.59)	725 (51.27)	< 0.001
Age (years)	62.6 ± 13.4	64.9 ± 14.9	0.505
Surgery, n (%)			< 0.001
Cataract	1305 (54.44)	669 (47.31)	
Glaucoma	557 (23.24)	402 (28.43)	
Pars plana vitrectomy	535 (22.32)	343 (24.26)	

The included patients were those who underwent primary vitrectomy surgery, glaucoma surgery or cataract surgery with monocular diseases. The primary vitrectomy surgery was performed in patients with retinal detachment, proliferative vitreoretinopathy and proliferative diabetic retinopathy. Glaucoma surgery was performed in patients with glaucoma diseases. Cataract surgery should be performed in patients with indications according to the latest Preferred Practice Pattern (PPP) for adult cataract surgery (2016). We excluded the patients with ocular trauma and infectious endophthalmitis.

### Perioperative treatment

The preoperative examinations included slit lamp biomicroscopy, direct and indirect ophthalmoscopy, visual electrophysiology, corneal endothelium counting, optical coherence tomography (OCT), ultrasound biomicroscopy, and intraocular pressure (IOP) measurement.

For the patients who did not receive tobramycin-dexamethasone eye ointment, the surgeries were performed by the experienced surgeons (supplemental Table 1). In contrast, for the patients who received tobramycin-dexamethasone eye ointment at the end of surgery, the surgeries were performed by the same experienced surgeons from the Department of Ophthalmology of Tianjin Medical University General Hospital (supplemental Table 1). At the end of surgery, 1–1.5-cm-long of tobramycin-dexamethasone eye ointment (Alcon-Couvreur SA, Puurs, Belgium) was applied in the conjunctival sac.

The preparation before surgery was standardized for all cases, including using antibiotic eye drops four times per day for at least 3 days before surgery. The patient’s eyelashes were cut before pars plana vitrectomy (PPV). The iodine wipe area included the eyelids and adjacent skin on the nose, cheek, forehead, and temporal area. The head was wrapped in a sterilized cloth and the patient was covered with a sterilized sheet. It was worth noting that povidone iodine was used for conjunctival sac disinfection during surgery. All patients underwent standard surgery by the same team of surgeons. After surgery, the patients returned to the ward. The patients only treated with tobramycin-dexamethasone four times per day for a week. No other types of antibiotics were used intraoperatively for any patients.

### Follow-up

At our center, ophthalmology examinations are routinely carried out at 1, 3, 5, and 7 days after surgery, including visual acuity, slit lamp biomicroscopy, direct ophthalmoscopy, and intraocular pressure and the forward reaction were recorded. The complaints of patients after the surgery were also recorded it. After discharge, the patients are followed once a year or if needed.

### Bacterial detection

In order to detect whether there was bacterial contamination after the opening of a tobramycin-dexamethasone eye ointment tube, bacterial culture was performed, according to the previous method [18]. Briefly, Tobramycin-dexamethasone eye ointment was obtained by the gentle application of a sterile swab at 30 min, 1 h, 1.5 h, 2 h, 2.5 h, 3 h, 6 h, 8 h, 24 h, 36 h, 48 h, 72 h, and 7 d after the ointment was opened and did not touch the cap or body of the bottle. Then apply the obtained ointment on a slide to preform the bacterial culture and Giemsa staining. After incubated in a 37 °C constant temperature CO_2_ culture solid medium for 24 h, we observed the bacterial growth. Bacterial strain was recorded by microbial automatic identification system.

### Statistical analysis

All data were analyzed using SPSS 16.0 (IBM, Armonk, NY, USA). Continuous data were tested for normal distribution using the Kolmogorov-Smirnov test. The results are presented as means ± standard deviation (SD), and were analyzed using the Student t test. Categorical data are presented as frequencies, and were analyzed using the chi-squared test. The differences were considered statistically significant at *P* < 0.05.

## Results

### Characteristics of the patients/eyes

The eye ointment group was composed of 997 males and 1400 females, with a mean age of 62.6 ± 13.4 years (Table 1). The non-ointment group was composed of 725 males and 689 females, with a mean age of 64.9 ± 14.9 years. There was no significant difference between the two groups regarding age (*P* = 0.505). The eye ointment treatment group included 1305 eyes treated for cataract, 535 eyes treated with vitrectomy, and 557 eyes treated for glaucoma. The non-ointment treatment group included 669 eyes treated for cataract, 343 eyes treated with vitrectomy, and 402 eyes treated for glaucoma.

### Endophthalmitis occurrence

The mean follow-up was 2.2 years (range, 1 to 4 years). Three patients suffered postoperative endophthalmitis (Table 2), and all of them were in the eye ointment group (3/2397, 0.13%) and were with a primary diagnosis of cataract and glaucoma. The diagnosis of endophthalmitis was standard and included obvious pain, a sudden drop in vision and increased conjunctival sac secretions, as well as vitreous cavity turbidity and anterior chamber empyema as evidenced on B-mode ultrasonography. These three patients with endophthalmitis were followed for 3 years. There was no significant difference in the occurrence of postoperative endophthalmitis between the eye ointment group and the non-ointment group (*P* = 0.184).

**Table 2:** Anterior chamber reaction and patient complaints

Variable	Eye ointment group (n = 2397)	Non-ointment group (n = 1414)	P
One day postoperatively, n (%)			
> 1+ cell	163 (6.80)	129 (9.12)	0.009
Corneal edema	174 (7.26)	142 (10.04)	0.003
Eye secretions increase	784 (32.71)	167 (11.81)	< 0.001
Foreign body feeling	938 (39.13)	157 (11.10)	< 0.001
One month postoperatively, n (%)			
> 0 cell	26 (1.08)	13 (0.92)	0.624
Corneal edema	26 (1.08)	13 (0.92)	0.624
Postoperative endophthalmitis, n (%)	3 (0.13)	0	0.184

As shown in Table 2, the anterior chamber reactions 1 day after surgery were more serious in the eye ointment group compared with the non-ointment group (all *P* < 0.05). Consequently, patient complaints such as increased eye secretions and a foreign body feeling were more frequent postoperatively in the eye ointment group (all P < 0.05), but there were no statistically significant differences at 1 month postoperatively (all *P* > 0.05).

### Eye ointment tube contamination

The tobramycin-dexamethasone eye ointment was collected after opening the tube and cultured for bacterial contamination. The contamination rate was 0% at all time points over 7 days.

## Discussion

There are no data available regarding the complications associated with using antibiotic ointment at the end of intraocular surgery. Therefore, this study aimed to explore the necessity of using ocular tobramycin-dexamethasone prophylactically at the end of intraocular surgery. The results suggest that prophylactic tobramycin-dexamethasone eye ointment did not decrease the risk of endophthalmitis after intraocular surgery. Preoperative antibiotics and standardized surgical disinfection procedures probably play important roles in the prevention of endophthalmitis.

Topical antibiotics are used at after intraocular surgery to prevent pathogenic microbial invasion and prevent bacterial endophthalmitis, and are considered as routine in almost all eye surgeries. The most common cause of endophthalmitis is Gram-positive bacteria and coagulase-negative cocci, which come from the eyelids, conjunctiva, lacrimal glands, hands of surgeons, nurses and anesthesiologists, surgical instruments, suction pipe perfusion, flushing fluid, vitreous substitutes, eye ointment after surgery, and operating room air [19, 20]. Regarding the prophylactic measures based on povidone-iodine 10% solution for lids and lashes, during small-gauge vitrectomy, the conjunctiva is displayed before making a trocar entry, giving rise to patient complaints of pain and blurred vision, but following PPV, endophthalmitis can be reduced [21]. There are no reported data illustrating the correlation between the occurrence of endophthalmitis and the use of topical antibiotics immediately following intraocular surgery.

In the present study, tobramycin-dexamethasone eye ointment use did not decrease the occurrence of endophthalmitis. Furthermore, patient complaints such as eye secretions and a foreign body feeling were increased in the tobramycin-dexamethasone eye ointment group compared with the non-ointment group. Therefore, these results suggest that the use of topical tobramycin-dexamethasone eye ointment at the end of intraocular surgery is unnecessary, but provided that a strict protocol is followed. First, preoperative antibiotics were used for the prevention of acute endophthalmitis. Second, sufficient and entire preoperative disinfection of the eyelid was performed. In addition, povidone iodine was used for conjunctival sac disinfection during surgery. Moreover, the eye ointment routinely used at our center contains tobramycin and dexamethasone, which may have caused an allergic reaction in the eye. Though the conjunctival and corneal incisions were tightly sutured after intraocular surgery, there were still some gaps in the incision, which caused a siphoning action of the ointment accompanied by hypotension in the eye. Of course, there is the possibility of bacterial resistance to antibiotics developing over time [21]. Furthermore, the cost of antibiotics is part of the medical burden in China. In the process of opening the ointment bottle, it is possible to smear some ointment in some cases, which can transport bacteria into the eyeball through the incision. In the operating room, one ointment tube was usually used for several patients in one day, which can also affect the sterility of the eye ointment.

High-quality evidence about preoperative antibiotics for the prevention of acute endophthalmitis after cataract surgery has been reported in the Cochrane Library, offering different suggestions about the use of topical antibiotics [22]. One report showed that an injection of cefuroxime with or without topical levofloxacin can decrease the chance of endophthalmitis after surgery [23]. Another suggested that the use of antibiotic eye drops in addition to an antibiotic injection reduced the chance of endophthalmitis compared with using an injection or eye drops alone [24]. Therefore, it is questionable whether to use topical antibiotics prophylactically. Topical antibiotics used before or after an ocular surgery for avoiding endophthalmitis are controversial, as harmful effects have been highlighted with the use of topical antibiotics, such as increased antibiotics resistance.

Prophylactic antibiotic use in intraocular surgery has rarely been reported, and is usually discussed in injection surgery. The reported rate of endophthalmitis following cataract surgery and intravitreal injections was 0.07–0.4% and 0.038–0.065%, respectively [25]. Surgical risk factors associated with endophthalmitis after PPV include inadequate wound closure, a postoperative hypotonic state, vitreous incarceration at a sclerotomy site, and aqueous intraocular tamponade. The related factors for antibiotic regimens are training (60.1%) and personal experience (58.1%), which was reported by a survey study that comprised 782 oculoplastic surgeons in 43 countries [26]. The occurrence of endophthalmitis is often related to the patients’ own condition; patients who are immunocompromised or taking preoperative topical steroids often have an elevated risk of endophthalmitis after PPV [27].

To date, few clinical studies have focused on bacterial detection by culturing the tobramycin-dexamethasone eye ointment tube after it was opened. The present study provides a chronological report of the pathogens in the eye ointment tube after being opened. All tested samples over 7 days were negative, but it cannot be ruled out that there are bacteria in tobramycin-dexamethasone eye ointment after being opened. A detection rate of positive bacteria in eye ointment of 30–50% has been reported by several studies [28, 29]. Therefore, another implication of the present study is that doctors should be cautious regarding the use of tobramycin-dexamethasone eye ointment at the end of intraocular surgery, as well as the consequence of increasing the risk of endophthalmitis after surgery. In the future, attention should be paid to the cautious use of antibiotic eye drops at the end of intraocular surgery.

Of course, the present study has limitations. The sample size was relatively small and from a single hospital. Only one doctor systematically used the tobramycin-dexamethasone eye ointment at the end of intraocular surgery, while the others did not, probably introducing some bias. Nevertheless, the entire preoperative and intraoperative disinfection protocol is the same for all surgeons. Finally, the occurrence of endophthalmitis events was very low, preventing any analysis of the factors associated with the occurrence of endophthalmitis.

In conclusion, using tobramycin-dexamethasone eye ointment did not decrease the risk of endophthalmitis after intraocular surgery, although there was not sufficient evidence to demonstrate that it is better not to use topical antibiotics (such as other kinds of antibiotic eye drops or ointment) at the end of intraocular surgery. Considering antibiotics resistance, surgical treatment specifications, side effects of the ointment, increased eye secretions, uncomfortable feeling, and cost burden, we suggest that it is not necessary to use tobramycin-dexamethasone eye ointment prophylactically at the end of intraocular surgery, but prospective trials will be necessary to confirm those results. In the future study we plan to perform the large sample randomized controlled trial with a non-inferiority design to confirm our conclusion.

## Conclusions

We did not observe a statistically significant difference in the incidence of endophthalmitis in patients with or without prophylactic tobramycin-dexamethasone eye ointment. And tobramycin-dexamethasone eye ointment seemed to increase some side effects such as eye secretions increasing and foreign body feeling.

## Supplementary information

**Additional file 1: Table S1.** Contributions of each surgeon.

## Data Availability

The datasets used and/or analyzed during the current study available from the corresponding author on reasonable request.

## Abbreviations

PPP: Preferred Practice Pattern
OCT: Optical coherence tomography
IOP: Intraocular pressure
PPV: Pars plana vitrectomy
