# Supplementary material for: Is it necessary to use tobramycin-dexamethasone eye ointment prophylactically in eyes at the end of intraocular surgery?
Source: BMC Ophthalmol. 2020 May 27;20:208. doi: 10.1186/s12886-020-01476-z (PMC7254748; doi:10.1186/s12886-020-01476-z)
Supplement: Supplementary file 1 — Additional file 1: Table S1. Contributions of each surgeon. [file 12886_2020_1476_MOESM1_ESM.docx]

**Supplemental Table1. Contributions of each surgeon.**

|  | Eye ointment group  (n=2397) | | | Non-ointment group  (n=1414) | | |
| --- | --- | --- | --- | --- | --- | --- |
| Surgery,  n (%) | Cataract | Glaucoma | Pars plana vitrectomy | Cataract | Glaucoma | Pars plana vitrectomy |
|  |  |  |  |  |  |  |
| Doc.1 | 43 | 0 | 0 | 26 | 0 | 0 |
| Doc.2 | 529 | 0 | 0 | 274 | 0 | 0 |
| Doc.3 | 0 | 25 | 0 | 0 | 17 | 0 |
| Doc.4 | 182 | 61 | 185 | 113 | 52 | 116 |
| Doc.5 | 77 | 127 | 0 | 56 | 94 | 0 |
| Doc.6 | 53 | 91 | 194 | 56 | 67 | 138 |
| Doc.7 | 76 | 28 | 0 | 38 | 21 | 0 |
| Doc.8 | 32 | 0 | 0 | 7 | 0 | 0 |
| Doc.9 | 313 | 225 | 156 | 99 | 151 | 89 |
